# Adaptor Complex 2 Controls Dendrite Morphology via mTOR-Dependent Expression of GluA2

**DOI:** 10.1007/s12035-017-0436-3

**Published:** 2017-02-11

**Authors:** Alicja Koscielny, Anna R. Malik, Ewa Liszewska, Justyna Zmorzynska, Aleksandra Tempes, Bartosz Tarkowski, Jacek Jaworski

**Affiliations:** Laboratory of Molecular and Cellular Neurobiology, International Institute of Molecular and Cell Biology, Ks. Trojdena St. 4, 02-109 Warsaw, Poland

**Keywords:** AP2b1, GluA2, Dendritic arbor, mTOR

## Abstract

The formation of dendritic arbors in neurons is a highly regulated process. Among the regulators of dendritogenesis are numerous membrane proteins that are eventually internalized via clathrin-mediated endocytosis. AP2 is an adaptor complex that is responsible for recruiting endocytic machinery to internalized cargo. Its direct involvement in dendritogenesis in mammalian neurons has not yet been tested. We found that the knockdown of AP2b1 (β2-adaptin), an AP2 subunit, reduced the number of dendrites in developing rat hippocampal neurons and decreased α-amino-3-hydroxy-5-methyl-4-isoxazolepropionic acid (AMPA) receptor subunit GluA2 levels by inhibiting mechanistic/mammalian target of rapamycin (mTOR). The dendritic tree abruption that was caused by AP2b1 knockdown was rescued by the overexpression of GluA2 or restoration of the activity of the mTOR effector p70S6 kinase (S6K1). Altogether, this work provides evidence that the AP2 adaptor complex is needed for the dendritogenesis of mammalian neurons and reveals that mTOR-dependent GluA2 biosynthesis contributes to this process.

**Electronic supplementary material:**

The online version of this article (doi:10.1007/s12035-017-0436-3) contains supplementary material, which is available to authorized users.

## Introduction

Classes of neurons with different functions have unique shapes of dendritic arbors to ensure the accurate responses of neurons to information they receive [1]. The multistep process of dendritic tree development is tightly regulated by various extracellular and intracellular factors [2–4], but cross-talk between various factors during dendritogenesis has been scarcely investigated.

Clathrin-mediated endocytosis (CME) enables (*i*) a variety of molecules to enter a cell upon ligand–receptor interactions, (*ii*) the control of signal transduction that is initiated by these interactions, and, (*iii*) in the case of neurons, the control of synaptic transmission. In CME, the intake of cargo occurs via membranous clathrin-coated pit with the help of a wide range of accessory adaptor proteins, including the AP2 adaptor complex [5]. AP2 is composed of four subunits: AP2a1 (α1-adaptin)/AP2a2 (α2-adaptin), AP2b1 (β2-adaptin), AP2m1 (adaptin-μ2), and AP2s1 (σ2-adaptin). These subunits bind cargo (AP2b1 and AP2m1), the plasma membrane (AP2a1/2 and AP2m1), clathrin, and other accessory proteins (e.g., Eps15 and AP180 [AP2b1]) [6]. CME is utilized by several receptors that regulate nervous system development and physiology, including α-amino-3-hydroxy-5-methyl-4-isoxazolepropionic acid (AMPA) receptors [7–9]. Consequently, CME contributes to neuronal development (e.g., dendritic growth and pruning; [10–14] and synaptic plasticity [9]). However, the particular role of AP2 during dendritogenesis has been scarcely investigated [12] and, to date, evidence is still lacking concerning the role of AP2 in mammalian dendritogenesis.

AMPARs mediate fast excitatory transmission in glutamatergic synapses in mammals. In the hippocampus, AMPARs mainly consist of GluA1-GluA2 and GluA2-GluA3 heterotetramers that can undergo dynamic and fast redistribution in and out of the plasma membrane, a process that contributes to synaptic plasticity in mature neurons [15–17]. AMPARs interact with AP2. Upon long-term depression (LTD) induction or the application of *N*-methyl-D-aspartate (NMDA), AMPARs undergo endocytosis, which requires this adaptor complex [9]. Less is known about the trafficking and function of AMPARs in developing neurons. Earlier studies demonstrated the importance of AMPARs for the growth of apical dendrites in developing rat cortical neurons in organotypic in vitro cultures [18] and the experience-dependent dendritic arbor growth of tectal neurons in *Xenopus laevis* in vivo [19].

One important regulator of dendritic arborization is mechanistic/mammalian target of rapamycin (mTOR; [20–22]). mTOR is a protein kinase that is known to adjust intercellular processes in response to extracellular factors [23, 24]. In neurons, mTOR complex 1 (mTORC1) plays a role in the control of protein translation [24, 25]. However, the specific proteins that are translated in an mTORC1-dependent fashion and contribute to dendritogenesis remain unknown. Also, the link between CME and mTORC1 in dendritogenesis has not yet been thoroughly studied. The present study provides evidence that this link indeed exists and plays an important role in regulating the levels of GluA2 in neurons and dendritic arborization. Specifically, AP2b1 knockdown led to a reduction of the number of dendrites in developing rat hippocampal neurons in vitro and in vivo. AP2 deficiency also resulted in a decrease in the cellular levels of GluA2, which was not an effect of a disturbance in membrane trafficking or the accelerated degradation of this protein. Instead, our data indicated that the inhibition of mTOR-controlled GluA2 expression was a likely cause of the decrease in GluA2 levels upon AP2 knockdown. Finally, we found that dendritic tree abruption that was caused by AP2 knockdown was rescued by the overexpression of functional GluA2 subunits or restoration of the activity of the downstream mTORC1 effector p70S6 kinase (S6K1).

## Materials and Methods

### Antibodies and Reagents

The primary antibodies that were used in the present study are listed in Table 1. Alexa Fluor 488-, 568-, 594-, and 647-conjugated secondary antibodies (anti-mouse, anti-goat, and anti-rabbit) were purchased from Invitrogen (Carlsbad, CA). Horseradish peroxidase-conjugated secondary antibodies and anti-mouse/anti-rabbit IRDye® 680RD and IRDye® 800CW were obtained from Jackson ImmunoResearch (West Grove, PA) and LI-COR Biosciences (Lincoln, NE), respectively. Rapamycin and cycloheximide were purchased from Calbiochem (La Jolla, CA) and Sigma-Aldrich (St. Louis, MO), respectively. NMDA, brain-derived neurotrophic factor (BDNF), 6-cyano-7-nitroquinoxaline-2,3-dione (CNQX), and D-2-amino-5-phosphonopentanoate (AP5) were purchased from Sigma-Aldrich (St. Louis, MO). AMPA was obtained from Tocris Bioscience (Minneapolis, MN).

**Table 1:** Primary antibodies used for the study

Antigen	Manufacturer	Catalog no.	Host	Application	Dilution
CD63	Santa Cruz Biotechnology, Dallas, TX	H-193	Mouse	IF	1:100
GFP	Roche Applied Science, Penzberg, Germany	11814460001	Mouse	WB	1:5000
GluA1	EMD Millipore, Billerica, MA	AB1504	Rabbit	WB	1:1000
GluA2	EMD Millipore	MAB397	Mouse	WB, IF	1:500, 1:100
Phospho-4E-BP1 (Thr37/46)	Cell Signaling Technology, Danvers, MA	2855	Rabbit	WB	1:250
Phospho-p70 S6 kinase (Thr389)	Cell Signaling Technology	9206	Mouse	WB	1:1000
Rab7	Cell Signaling Technology	D9572 XP	Rabbit	IF	1:100
RFP	Abcam, Cambridge, UK	62341	Rabbit	IHC	1:250
TrkB	Cell Signaling Technology	4603	Rabbit	WB	1:1000
α-Adaptin	BD Biosciences, Franklin Lakes, NJ	610502	Mouse	WB	1:1000
α-tubulin	Sigma-Aldrich, St. Louis, MO	T5168	Mouse	WB	1:10,000
β2-Adaptin (AP2b1)	Santa Cruz Biotechnology	6435	Goat	WB, IF	1:1000 1:100
β-Galactosidase (β-Gal)	Promega, Madison, WI	Z3781	Mouse	WB	1:1000

*IF* immunofluorescence, *IHC* immunohistochemistry, *WB* Western blot

### DNA Constructs

The following mammalian expression plasmids were described previously: pSuper [26], β-actin-GFP [20], β-actin-mRFP [27], EFα-β-gal [28], pEGFP-C1 (Clontech, Mountain View, CA), pGW1 (British Biotechnology, UK), pRK5-p70S6KT389E (pS6K^ca^; [29]), pSuper-GluA2sh [30], pSuper-shAP2b1#1, pSuper-shAP2b1#2, pSuper-shAP2b1#3 [31], pCMV-VSV-G [32], RSV-Rev, and pMDLg/pRRE [33]. pUltra-Chili (Addgene plasmid no. 48687) was a gift from Malcolm Moore. Plasmids that encoded GluA2 and its mutants GluA2ΔC, GluA2Q566R, and GluA2Q581R were kind gifts from Dr. M. Passafaro [30, 34] and were used to subclone GluA2-encoding sequences to the pGW1 vector. Short-hairpin RNAs (shRNAs) against rat α-adaptin (GenBank no. NM_001107511) were designed to target the following sequences: 5′-TCCGGCTCATTAACAACGCTATC-3′ (shAP2a1#1), 5′-TCCAGGACTACACTTACTACTTC-3′ (shAP2a1#2), and 5′-CGGAGACAGCATGGATAGTGTGA-3′ (shAP2a1#3) and inserted into pSuper. Scrambled shRNAs were designed based on the original AP2b1 shRNA sequences using the online siRNA Wizard v3.1 tool and cloned into the pSuper vector. The following sequences were used: 5′-GTCTCACAACAAGATATGT-3′ (scrAP2b1#1), 5′-GATCATTACCCGTACTATA-3′ (scrAP2b1#2), and 5′-GGGTGAGATCATGAGAGAA-3′ (scrAP2b1#3). shAP2b1#2 was additionally subcloned to pUltra-Chili to obtain pUltra-Chili-shAP2b1. pEGFP-AP2b1 was obtained by subcloning rat AP2b1 cDNA into EcoRI/SalI sites of pEGFP-C1. pEGFP-AP2b1*, a plasmid that encodes the shRNA#2-resistant mutant of AP2b1, was generated from pEGFP-AP2b1 by introducing silent mutations in the shRNA target region using the QuikChange Site-Directed Mutagenesis System (Stratagene, Santa Clara, CA) with the following primers: 5′-GATCAGTGAGTCTCACCCAAACAGTAACCTGCTCGACTTGAACCCTCAGAATATC -3′ and 5′-GATATTCTGAGGGTTCAAGTCGAGCAGGTTACTGTTTGGGTGAGACTCACTGATC -3′. pGW1-GluA2ΔSP was obtained by deleting the N-terminal signal peptide (MQKIMHISVLLSPVLWGLIFGV) from the wild-type GluA2 construct according to the protocol of the Q5® Site-Directed Mutagenesis Kit (New England Biolabs, Ipswich, MA) using the following primers: 5′-CATTTCCAAGAA-AAGTAGAGCATAAG-3′ and 5′-TACCCATACGACGTCCCAGACTAC-3′.

### Lentiviral Production and Purification

For lentiviral vector production, the pUltra-Chili plasmid or its derivatives that encoded shRNA were co-transfected to HEK293T cells with third-generation packing system plasmids (pCMV-VSV-G, pRSV-Rev, and pMDLg-pRRE) using the calcium phosphate method. After 48 h, the medium from HEK293T cells was filtrated (0.22 μm pore size), aliquoted, and stored at −80 °C until needed for the in vitro culture experiments. For in vivo lentiviral vector injections, viral particles after production in HEK293T cells were additionally purified and concentrated. To accomplish this, medium from HEK293T cells was spun for 5 min at 1600×*g* at 4 °C, and the supernatant was filtered using a Filtermax rapid bottle filter (0.22 μm pore size; TPP Techno Plastic Products AG, Trasadingen, Switzerland). The medium was then centrifuged at 110,000×*g* at 4 °C for 90 min, and the pellet was dissolved in cold phosphate-buffered saline (PBS) that contained 10 % glycerol with shaking at 4 °C for 3.5 h. The dissolved pellet was quickly spun for 30 s at 14,000×*g*, and the supernatant was aliquoted and stored at −80 °C.

### Neuron Culture, Transfection, and Transduction

To obtain neurons for the tissue culture, pregnant female rats and rat embryos of both sexes were sacrificed according to a protocol that was approved by the 1st Ethical Committee in Warsaw, Poland (decision no. 288/2012), which was in compliance with European Community Council Directive 86/609/EEC. Primary hippocampal and cortical cultures were prepared from embryonic day 19 rat brains according to the procedure of Banker and Goslin [35] with modifications and transfected on day in vitro (DIV) 0 (cortical) or DIV7 (hippocampal) using Amaxa nucleofection or Lipofectamine 2000 (Invitrogen), respectively, as described previously [36]. For knockdown experiments, hippocampal neurons were co-transfected with either pSuper or pSuper that encoded the desired shRNA (or their scrambled counterparts), EFα-β-gal, and β-actin-GFP/β-actin-mRFP in a 1:1:1 ratio. When shRNA mix was used instead of single shRNA, it was co-transfected with β-actin-GFP in a 2:1 ratio. For the rescue experiments, neurons were co-transfected with pSuper-shAP2b1#2 or control pSuper, β-actin-GFP, and one of the following rescue constructs: pEGFP-C1-AP2b1*, pGW1-GluA2, pGW1-GluA2ΔC, pGW1-GluA2ΔSP, pGW1-GluA2Q566R, pGW1-GluA2Q581, or EFα-β-gal. The cells were fixed 72 h after transfection. Lentiviral vectors were produced as described above, and the resulting filtered supernatant that contained viral particles was added to DIV4 neurons for 5 h (30 μl/well in a 24-well plate) in the mix of fresh Neurobasal® medium (Thermo Fisher Scientific, Waltham, MA, USA) supplemented with 2 % B27 (Thermo Fisher Scientific), 1 % penicillin-streptomycin (Sigma-Aldrich), 0.5 mM glutamine (Thermo Fisher Scientific), and conditioned culture medium (1:1). After incubation with the viruses, the neurons were washed with non-supplemented Neurobasal® medium and further cultured in the mix of fresh Neurobasal® medium (supplemented as described above) and conditioned culture medium (1:1).

### Cell Line Culture and Transfection

RAT2 cells were grown in Dulbecco’s modified Eagle’s medium (DMEM) that contained 10 % fetal bovine serum and 1 % penicillin-streptomycin (Sigma-Aldrich) at 37 °C in a 5 % CO_2_ atmosphere. For the knockdown experiments, the cells were co-electroporated, using CUY21 device (Nepagene, Chiba, Japan) according to the manufacturer’s protocol, with either pSuper or pSuper-shAP2b1#2 and pEGFP-C1 in a 3:1 ratio. After electroporation, the cells were cultured for 48 h in DMEM with 2 % FBS at 37 °C in a 5 % CO_2_ atmosphere.

### Drug Treatment and Western Blot Analysis

Rapamycin (100 nM) was added to DIV7 hippocampal neurons for 48 h. Cycloheximide (10 μM) was delivered to hippocampal neurons 3 days post-transduction for 24 h. The collected cell lysates were analyzed by Western blot. For quantitative analysis, secondary antibodies conjugated to appropriate IRDyes® and the Odyssey Infrared Imaging System (LI-COR Biosystems) were used. The results were quantified using Image Studio™ Lite (LI-COR Biosystems). GluA2 degradation was measured as the ratio of GluA2 levels in cycloheximide (CHX)-treated cells to control cells in neurons that were transduced with Lv-Chili-shAP2b1 compared with Lv-Chili-infected neurons.

### Immunofluorescent Labeling of Hippocampal Neurons

Neurons were fixed using 4 % paraformaldehyde/4 % sucrose in PBS (137 mM NaCl, 2.7 mM KCl, 8 mM Na_2_HPO_4_, and 1.4 mM KH_2_PO_4_, pH 7.4) for 15 min at room temperature, washed three times with PBS, and incubated with primary antibody diluted in ADB buffer (1× PBS, 1 % bovine serum albumin [BSA], and 0.3 % Triton X-100) for GluA2 and CD63 staining or GDB buffer (30 mM phosphate buffer [pH 7.4], 0.2 % gelatin, 0.5 % Triton X-100, and 450 mM NaCl) for AP2b1 staining overnight at 4 °C. On the next day, the cells were washed three times with PBS and incubated with appropriate Alexa Fluor-conjugated secondary antibodies diluted 1:100 in ADB buffer for GluA2 and CD63 and 1:200 in GDB buffer for AP2b1 for 1 h at room temperature. Afterward, the cells were again washed with PBS, and coverslips were mounted using ProLong® Gold Antifade Reagent with or without DAPI (Cell Signaling Technology) or VECTASHIELD Antifade Mounting Medium (Vector Laboratories, Burlingame, CA).

### Transferrin Uptake Assay

Neurons and RAT2 cells were starved at 37 °C in a 5 % CO_2_ atmosphere in non-supplemented Neurobasal® medium or DMEM for 3 and 18 h, respectively. Alexa Fluor® 647-conjugated transferrin from human serum (Thermo Fisher Scientific) was then added to the cells for 5 and 10 min at a final concentration of 25 μg/ml. The cells were then rinsed with medium, fixed with paraformaldehyde, and washed with PBS. Afterwards, confocal images of cells were acquired and fluorescence intensity was analyzed as described below in the appropriate “Materials and Methods” section.

### GluA2 Internalization Assay

For the labeling of surface GluA2 receptors and subsequent analysis of their internalization, live neurons were incubated for 15 min at 37 °C in a 5 % CO_2_ atmosphere with anti-GluA2 primary antibody (10 μg/ml of conditioned culture medium), rinsed with warm Neurobasal® medium, and returned to the culture medium for an additional hour. For stimulation experiments, neurons were either returned to conditioned medium (control) or medium containing following drugs: 50 μM AMPA + 50 μM AP5, 50 μM NDMA + 50 μM CNQX, 100 nM BDNF, or 90 mM KCl for 3 min. The neurons were then fixed under non-permeabilizing conditions using ice-cold 4 % paraformaldehyde and 4 % sucrose in PBS for 7 min at room temperature and washed with PBS. The surface pool of anti-GluA2-bound receptor was detected by overnight incubation at 4 °C with a saturating amount of anti-mouse Alexa Fluor 647-conjugated secondary antibody (1:100) in ADB buffer without Triton X-100. On the next day, after washing in PBS, the cells were permeabilized with 0.3 % Triton X-100 in PBS for 5 min at room temperature and washed again with PBS. Following these steps, the intracellular pool of GluA2 was detected with anti-mouse Alexa Fluor 488- or 594-conjugated secondary antibody (1:100) in ADB buffer without Triton X-100 for 1 h at room temperature, followed by washing with PBS.

### Image Acquisition and Analysis

Images of fluorescently labeled in vitro cultured cells were acquired with a Zeiss LSM5 Exciter confocal microscope (×40 or ×63 oil objective) or Zeiss LSM710 NLO (×20 air or ×40 oil objective, ×1.5 zoom) at 1024 × 1024 pixel resolution. Z-stacks of the images were averaged twice per line, and the settings were kept constant for all of the scans. The Z-stacks were converted to single images using a maximum intensity projection. The fluorescent intensity was measured in soma and dendrites using ImageJ (National Institutes of Health, Bethesda, MD) integrated density or MetaMorph (Universal Imaging Corporation, Downingtown, PA) average intensity options, respectively. The colocalization Colormap plug-in for ImageJ was used for the quantification of colocalization [37]. For the morphometric analysis, images of transfected neurons were acquired using a Nikon TMS-F fluorescence microscope, and the total number of dendritic tips (TNDT) was counted manually using ImageJ Cell Counter plug-in. Images of lentiviral vector-transduced neurons in rat brain sections were obtained with a Zeiss LSM710 NLO confocal microscope using a ×20 objective at 1024 × 1024 pixel resolution. Z-stacks of the images were averaged twice per line. The obtained images were segmented semiautomatically, and the arbors were skeletonized using the Skeletonize 3D Fiji plug-in [38]. The skeletons were then analyzed by the 3D Sholl analysis Fiji plug-in [39]. The linear model (line on the graph) was fitted into empirical data (points on the graph), and the *R* ^2^ coefficient of determination was quantified. *R* ^2^ = 0.8 and 0.6 were treated as good and satisfactory fits, respectively.

### Total RNA Isolation for Quantitative Real-Time PCR

RNA from neurons was isolated using the RNeasy Mini Kit (Qiagen, Venlo, The Netherlands) according to the manufacturer’s protocol. cDNA was obtained with High Capacity RNA-to-cDNA Master Mix (Applied Biosystems, Foster City, CA, USA). Real-time quantitative polymerase chain reaction (RT-qPCR) was performed using the 7900HT Fast Real-Time PCR System (Applied Biosystems) and SYBR Green PCR Master Mix (Thermo Fisher Scientific) with the following primers: GAPDH (5′-CTGTTGAAGTCACAGGAGACA and 5′-CATCAAGAAGGTGGTGAAGCA; [40]) as the endogenous control and GluA2 (5′-CACTCTCGATGCCATATACGTTG and 5′-GCCGAGGCGAAACGAATGA; [41]) for the target gene. The data were acquired using the SDS 2.4 and RQ Manager 1.2.1 programs and analyzed using the 2(^−ΔΔCt^) method for relative quantification.

### In Vivo Lentiviral Injections and Preparation of Brain Slices

The procedure was performed according to a protocol that was approved by the 1st Ethical Committee in Warsaw, Poland (decision no. 674/2015). Neonatal Wistar rats (postnatal day 4 [P4], both sexes) were anesthetized on ice for 5 min. The animals were then placed on a support in a stereotaxic frame under a Hamilton syringe with a manually pulled glass capillary (<50 μm diameter). Afterward, 1–1.5 μl of the lentiviral vector solution was injected into the left and right hemispheres using the following coordinates: 2 mm medial/lateral, 3 mm anterior/posterior, and 2 or 3 mm dorsal/ventral from lambda. After the injection, the animals were allowed to recover on a heated pillow. Two weeks later, the rats were intraperitoneally (IP) anesthetized with 300 mg/kg pentobarbital and transcardially perfused with PBS-II (147 mM NaCl, 2.7 mM KCl, and 2 mM phosphate buffer, pH 7.4) and PFA-II (4 % PFA in 0.1 M phosphate buffer, pH 7.4). The brains were then isolated, postfixed overnight in PFA-II, and cryopreserved with 30 % sucrose in 0.1 M phosphate buffer for approximately 72 h. The brains were then cut into 100-μm-thick coronal sections using a Vibrating-Blade Microtome (HM 650V, Thermo Fisher Scientific) and collected in antifreeze medium (15 % sucrose, 35 % ethylene glycol, 25 mM phosphate buffer [pH 7.4], and 10 mM NaF). Prior to immunostaining, brain sections were washed with PBS-II and underwent antigen retrieval (10 mM trisodium citrate and 0.05 % Tween-20) for 15 min at 60 °C, followed by washing with PBS-II. The brain slices were then treated with blocking buffer (PBS-II supplemented with 1 % donkey serum and 1 % Triton-X-100) for 1 h at room temperature and incubated with α-RFP primary antibodies (1:250 in blocking buffer) overnight at 4 °C. Afterward, the slices were washed in PBS-II and incubated with Alexa Fluor 594-conjugated secondary antibodies (1:200 in blocking buffer) for 2 h at room temperature. The brain sections were then washed with PBS-II and counterstained with Hoechst 33258 (1 μg/ml, 1:10,000; Invitrogen).

### Statistical Analysis

The exact numbers of cells (*n*) and culture batches (*N*) that were examined for the respective experiments are provided in the figure legends. The statistical analyses were performed using Prism software (GraphPad, San Diego, CA). The data were analyzed using the Kruskal-Wallis test and Dunn’s multiple-comparison post hoc test or one-way ANOVA test followed by Bonferroni multiple-comparison test. For comparisons between two groups, the Mann-Whitney test or one-sample *t* test was used to verify statistical significance.

## Results

### AP2b1 Controls Dendritic Arbor Morphology

**Fig. 1:**
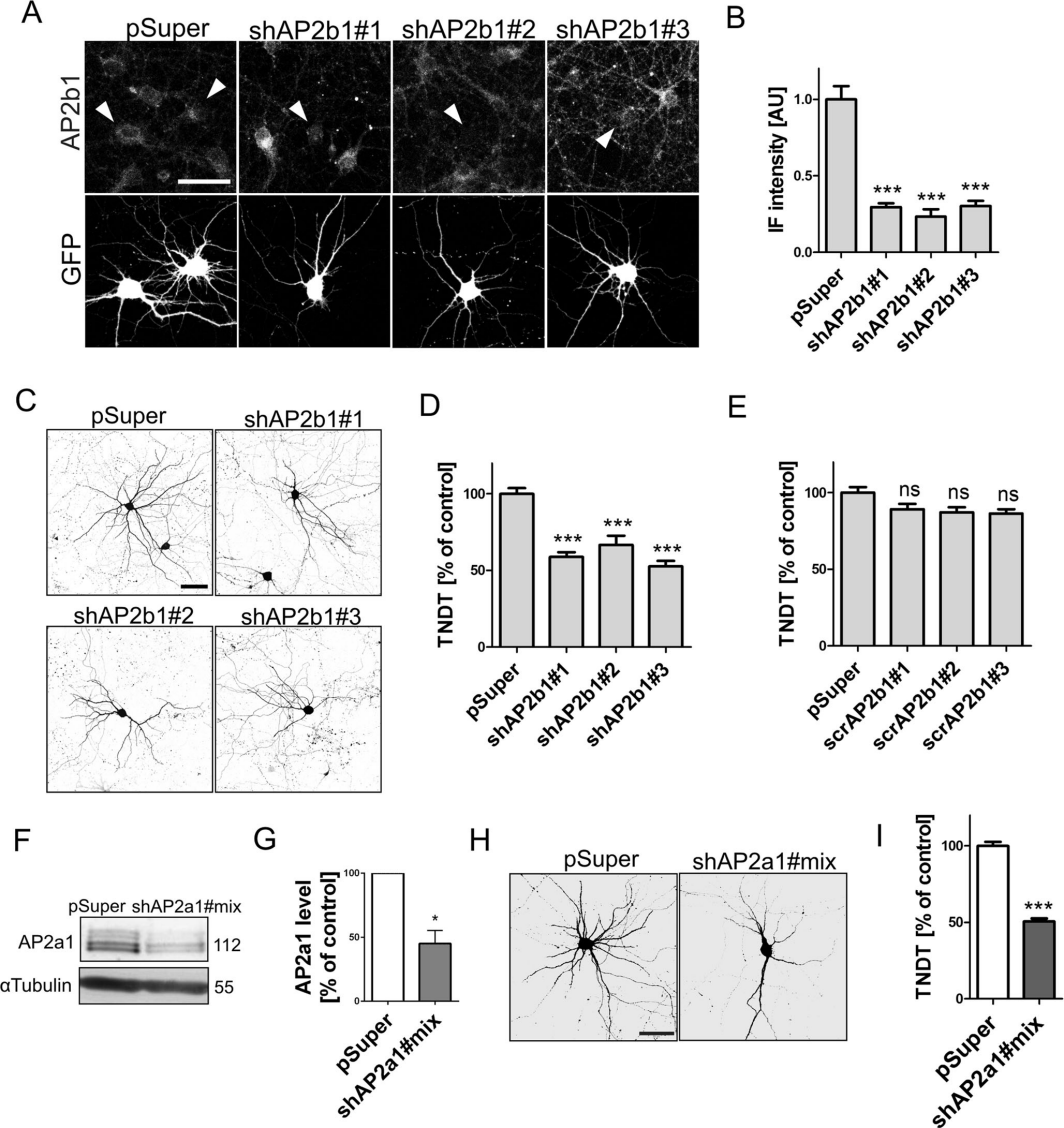
AP2 controls dendritic arbor morphology in neurons cultured in vitro. **a** Representative confocal images of DIV10 cultured hippocampal neurons immunofluorescently stained for AP2b1. Cells were transfected on DIV8 for 2 days as indicated. Neurons were additionally co-transfected with GFP-encoding plasmid for the identification of transfected cells (*arrowheads*). *Scale bar* = 50 μm. **b** Quantification of level of AP2b1 immunofluorescence in the cell body of neurons transfected as in **a**. The data are expressed as a mean value normalized to control ± SEM. ***p < 0.001 (Kruskal-Wallis test followed by Dunn’s multiple-comparison test). Cell images were obtained from two independent culture batches. Number of cells per variant (*n*): pSuper (22), shAP2b1#1 (23), shAP2b1#2 (18), and shAP2b1#3 (22). **c** Representative confocal images of cultured hippocampal neurons transfected on DIV8 for 4 days as indicated. Neuron morphology was visualized by co-transfection with GFP. *Scale bar* = 50 μm. **d** Total number of dendritic tips (TNDT) of neurons transfected as in **c**. The data are expressed as a mean value normalized to control ± SEM. ***p < 0.001 (Kruskal-Wallis test followed by Dunn’s multiple-comparison test). Cell images were obtained from two independent culture batches. Number of cells per variant (*n*): pSuper (36), shAP2b1#1 (36), shAP2b1#2 (17), and shAP2b1#3 (37). **e** TNDT of hippocampal neurons transfected on DIV8 for 4 days with a plasmids scrambled shRNAs against AP2b1. The data are expressed as a mean value normalized to control ± SEM. *ns*, nonsignificant (Kruskal-Wallis test followed by Dunn’s multiple-comparison test). Cell images were obtained from two independent culture batches. Number of cells per variant (*n*): pSuper (25), scrAP2b1#1 (20), scrAP2b1#2 (21), and scrAP2b1#3 (21). **f** Western blot analysis of α-adaptin (AP2a1) and tubulin in protein lysates obtained from cortical neurons nucleofected on DIV0 for 2 days with empty pSuper or a pool of pSuper plasmids that encoded shRNAs against α-adaptin. **g** Results of quantitative WB analysis of cell lysates obtained from neurons nucleofected as in **f**. *p < 0.05 (one-sample *t* test). Number of independent experiments (*N*) = 3. *Error bars* indicate SEM. **h** Representative confocal images of cultured hippocampal neurons transfected on DIV7 for 3 days as indicated. Neuron morphology was visualized by co-transfection with GFP-encoding plasmid. *Scale bar* =50 μm. **i** TNDT of neurons transfected as in **h**. The data are expressed as a mean value normalized to control ± SEM. ***p < 0.001 (Mann-Whitney test). Cell images were obtained from four independent culture batches. Number of cells per variant (*n*): pSuper (78) and shAP2a1#mix (75)

Although CME is important for mammalian dendritogenesis, the direct involvement of AP2 has not been studied to date. In the absence of AP2, the endocytosis of cargo can still occur via alternative entry routes. Therefore, we sought to verify the role of AP2 in dendritogenesis. First, we designed three shRNAs against rat AP2b1, cloned them into a pSuper vector, transfected them to DIV8 hippocampal neurons, and validated, using immunofluorescence, their efficacy against endogenous AP2b1 2 days later (Fig. 1a, b). The next series of experiments was performed to determine whether the lower amount of AP2b1 affects proper dendritic tree morphology in in vitro cultured neurons undergoing intensive dendritogenesis. DIV8 hippocampal neurons were transfected with plasmids encoding AP2b1 shRNAs, and the TNDT was counted after 4 days (Fig. 1c, d). As shown in Fig. 1d, the transfection of each AP2b1 shRNA lowered the TNDT compared with the pSuper control. In contrast, when we performed neuronal transfection using pSuper that encoded scrambled sequences of used shRNAs, we did not observe any significant changes in TNDT compared with control neurons (Fig. 1e). These results indicate the specific effects of the tested shAP2b1 shRNAs on the TNDT of developing neurons. The specificity of shAP2b1#2 was further confirmed by rescue experiments, in which DIV7 neurons were transfected with this particular shRNA and pEGFP-AP2b1* that encoded shAP2b1-resistant AP2b1 mRNA (EGFP-AP2b1*; Fig. 5e, f).

To further support the importance of AP2 for proper dendritic branching, we tested whether the knockdown of AP2a1 also leads to dendritic tree simplification. We first designed three shRNAs against AP2a1 and validated their efficiency in nucleofected neurons (Fig. 1f, g). Next, we transfected DIV7 hippocampal neurons with either pSuper or shAP2a1#mix, together with a GFP-encoding plasmid. Three days later, the TNDT quantification revealed that AP2a1 knockdown led to an approximately 50 % decrease in the TNDT (Fig. 1h, i). These results indicate that the AP2 is indeed required for shaping dendritic arbors in developing hippocampal neurons in vitro.

**Fig. 2:**
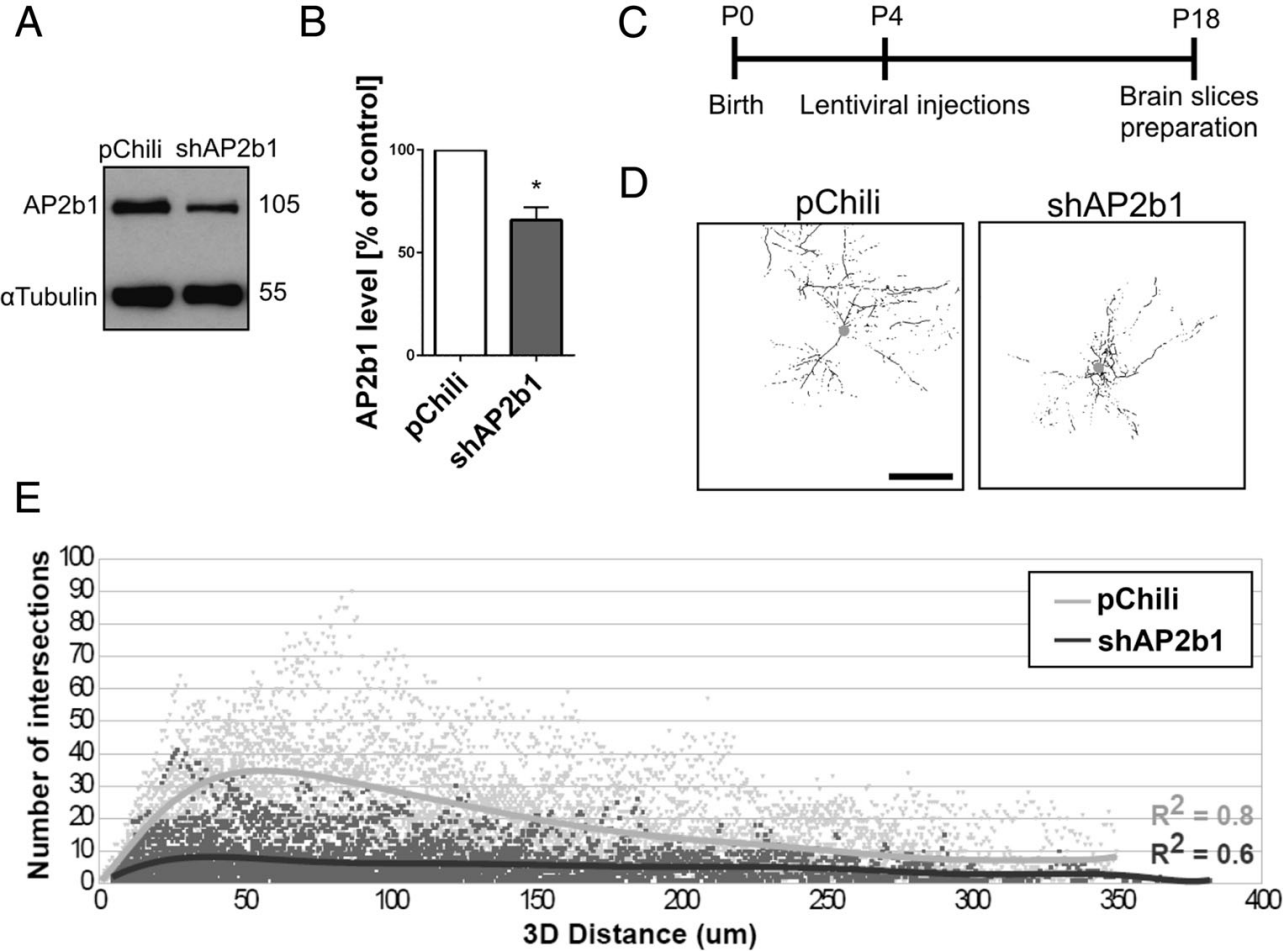
AP2 controls dendritic arbor morphology in vivo. **a** Western blot analysis of AP2b1 in protein lysates obtained from cultured hippocampal neurons infected with Lv-Chili or Lv-Chili-shAP2b1 on DIV4 for 6 days. **b** Results of quantitative WB analysis of cell lysates obtained from neurons transduced as in **a**. *p < 0.05 (one-sample *t* test). Number of independent experiments (*N*) = 4. *Error bars* indicate SEM. **c** The time-course of the experiment. **d** Representative 3D reconstructions of CA1 hippocampal neurons of rats transduced with Lv-Chili or Lv-Chili-shAP2b1 on P4 for 2 weeks. *Scale bar* = 100 μm. **e** Results of 3D Sholl analysis of CA1 hippocampal neurons from rats treated as in **b**. The data are expressed as the number of intersections as a function of the 3D distance from the soma. *R* ^2^ values = 0.8 for Lv-Chili (good fit) and 0.6 for Lv-Chili-shAP2b1 (satisfactory fit). Cell images were obtained from eight (Lv-Chili) or five (Lv-Chili-shAP2b1) animals. Number of cells per variant (*n*): Lv-Chili (28) and Lv-Chili-shAP2b1 (34)

To confirm the physiological relevance of our observations, we knocked down AP2b1 in developing hippocampal neurons in vivo. In all of the experiments described hereafter, including the in vivo experiments, we used shAP2b1#2 because of its best performance (Fig. 1a, b). We first engineered a lentiviral vector that simultaneously expressed AP2b1sh#2 and TdTomato (Lv-Chili-AP2b1sh), which effectively infected cultured hippocampal neurons and lowered their levels of AP2b1 (Fig. 2a, b). Next, Lv-Chili (control) and Lv-Chili-AP2b1sh were introduced to the hippocampi in P4 rats (Fig. 2c) for 2 weeks. Three-dimensional reconstruction and the Sholl analysis of transduced cells’ dendritic arbors (Fig. 2d, e) showed that the complexity of dendritic arbors of hippocampal neurons that were infected with Lv-Chili-AP2b1sh was decreased compared with control neurons that were infected with Lv-Chili.

### AP2 Is Needed for Endocytosis of Transferrin Receptor But Not GluA2 in Unstimulated Developing Neurons

**Fig. 3:**
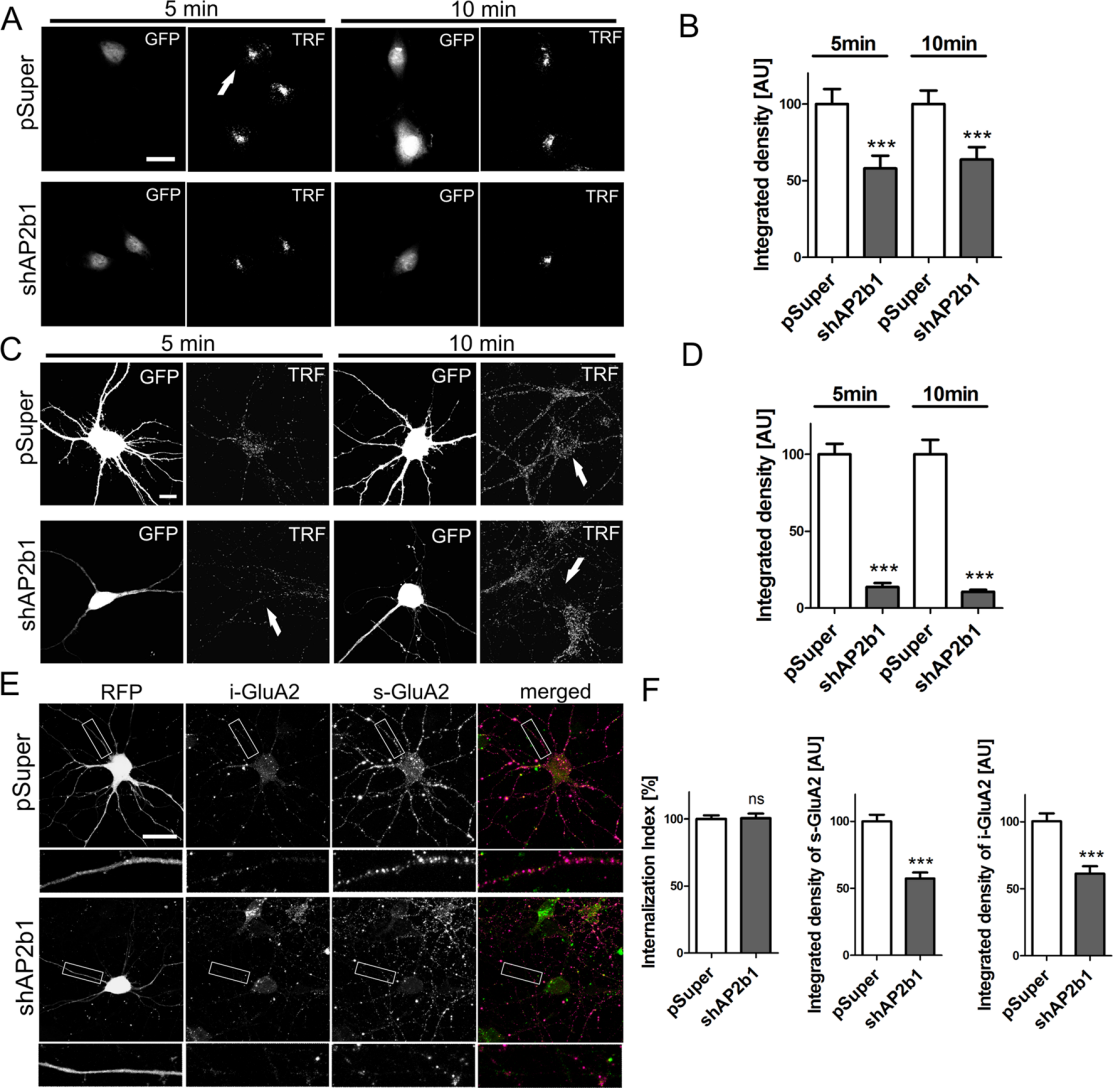
In developing neurons, AP2 contributes to endocytosis of transferrin receptor but not GluA2 under basal culture conditions. **a** Representative confocal images of RAT2 fibroblasts exposed to fluorescently labeled transferrin. Cells were electroporated for 2 days with empty pSuper or pSuper-shAP2b1#2 (shAP2b1) and pEGFP-C1 for the identification of transfected cells (*arrows*). After 2 days, the cells were starved overnight prior to 5 or 10 min fluorescently labeled transferrin uptake. *Scale bar* = 20 μm. **b** Quantification of integrated density of transferrin fluorescence in the cell body of RAT2 fibroblasts transfected and treated as in **a**. The data are expressed as a mean value normalized to control. *Error bars* indicate SEM. ***p < 0.001 (Mann-Whitney test). Cell images were obtained from four independent culture batches. Number of cells per variant (*n*): pSuper (55) and shAP2b1 (49) for 5 min uptake and pSuper (72) and shAP2b1 (52) for 10 min uptake. **c** Representative confocal images of cultured hippocampal neurons exposed to fluorescently labeled transferrin. DIV7 neurons were transfected (*arrows*) for 3 days as indicated. On DIV10, cells were starved for 3 h prior to 5 or 10 min fluorescent transferrin uptake. *Scale bar* = 10 μm. **d** Quantification of integrated density of transferrin fluorescence in the cell body of neurons transfected and treated as in **c**. The data are expressed as a mean value normalized to control. *Error bars* indicate SEM. ***p < 0.001 (Mann-Whitney test). Cell images were obtained from five independent culture batches. Number of cells per variant (*n*): pSuper (44) and shAP2b1 (52) for 5 min uptake and pSuper (50) and shAP2b1 (50) for 10 min uptake. **e** Representative confocal images of cultured hippocampal neurons transfected as indicated on DIV7 for 3 days and subjected to the GluA2 internalization assay. *i-GluA2*, internalized pool of GluA2; *s-GluA2*, surface pool of GluA2. *Scale bar* = 20 μm. **f** Quantitative analysis of integrated density of s-GluA2 and i-GluA2 dendritic fluorescence and GluA2 internalization index in neurons transfected and treated as in **e**. For s-GluA2 and i-GluA2, the data are expressed as a mean value normalized to control ± SEM. The internalization index was calculated using the following formula: *i-GluA2/s-GluA2 + i-GluA2*. The results are presented as a mean value normalized to control ± SEM. *ns*, nonsignificant; ***p < 0.001 (Mann-Whitney test). Cell images were obtained from five independent culture batches. Number of cells per variant (*n*): pSuper (50) and shAP2b1 (48)

After confirming that AP2 is required for the proper shape of dendritic arbors in hippocampal neurons, we next searched for the underlying mechanism. As a first step, we verified the effectiveness of shAP2b1#2 in inhibiting the CME of transferrin receptor [TfR], a prototypical AP2 cargo by performing a transferrin uptake assay in both non-neuronal cells (RAT2) and neurons. In the first series of experiments, RAT2 cells were electroporated with either pSuper or pSuper-shAP2b1#2 together with GFP for 48 h, and the RAT2 cells were serum-starved for 18 h prior to the addition of fluorescently labeled transferrin. The cells were fixed after 5 and 10 min of transferrin uptake, and the effectiveness of TfR CME was monitored by recording the fluorescence level of internalized transferrin (Fig. 3a). As shown in Fig. 3b, the transfection of shAP2b1#2 at both time points significantly decreased the amount of internalized transferrin compared with control cells. Next, we repeated analogous experiments using neurons that were transfected on DIV7 for 3 days with either pSuper or pSuper-AP2b1sh#2 (Fig. 3c). Similar to the effects of AP2 deficiency in RAT2 cells, AP2b1 knockdown in cultured neurons resulted in a significant reduction of transferrin uptake (Fig. 3d).

Several AP2 cargo (e.g., GluA2 and TrkB) contribute to dendritogenesis [18, 19, 42]. However, oppose to endocytosis of transferrin receptors, which is constitutive and AP2-dependent, internalization of these receptors occurs at low rate and in AP2-independent manner under basal culture conditions, at least in mature neurons [43, 44]. But role of AP2 in internalization of several such receptors in unstimulated, developing neurons cultured in vitro was not studied thus far. Therefore, we could not exclude a priori that disturbed internalization of AMPARs, for example, contributes to dendritic phenotype induced by AP2b1 knockdown in such cells. Thus, to rule out or confirm such possibility for AMPARs, we tested whether AP2b1 knockdown affects the CME of GluA2 in immature neurons under basal culture conditions. To do so, we performed a live internalization assay using neurons that were transfected on DIV7 for 3 days with pSuper or pSuper-shAP2b1#2, together with an RFP-encoding plasmid for the identification of transfected cells. The surface population of GluA2 (s-GluA2) was labeled in live cultured hippocampal neurons with an antibody against the extracellular portion of the receptor and then allowed to internalize GluA2 (i-GluA2) under basal culture conditions for 60 min (Fig. 3e). GluA2 internalization index in control cells and upon AP2b1 knockdown was the same (Fig. 3f), indicating that AP2b1 deficiency did not disturb basal GluA2 endocytosis in developing neurons. However, these experiments demonstrated that shAP2b1 knockdown decreased the levels of both the surface and internalized pool of GluA2 (Fig. 3f), suggesting a decrease in the total content of cellular GluA2. Given that GluA2 level is important for dendritogenesis of tectal and cortical neurons, we decided to focus on this particular finding.

### AP2 Regulates Cellular Levels of GluA2

**Fig. 4:**
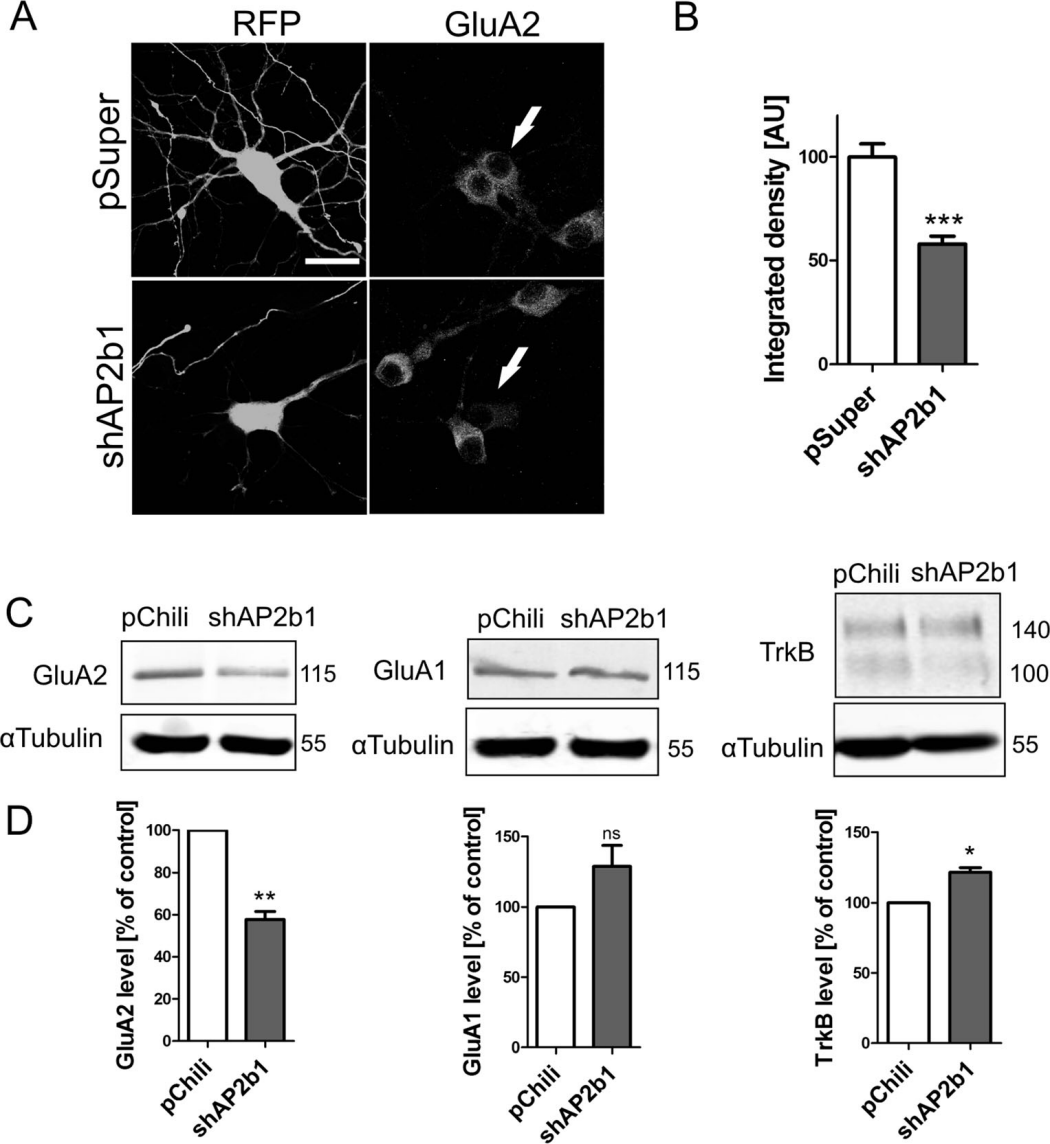
AP2 regulates cellular level of GluA2. **a** Representative confocal images of neurons transfected (*arrows*) as in Fig. 3e and immunofluorescently stained for GluA2. *Scale bar* = 20 μm. **b** Quantitative analysis of total level of GluA2 immunofluorescence in neurons transfected as in Fig. 3e. The integrated density of GluA2 immunofluorescence in cell bodies of transfected cells was normalized to that in non-transfected neurons. The data are expressed as a mean value normalized to control. *Error bars* indicate SEM. ***p < 0.001 (Mann-Whitney test). Cell images were obtained from six independent culture batches. Number of cells per variant (*n*): pSuper (48) and shAP2b1 (50). **c** Western blot analysis of GluA2, GluA1, and TrkB in protein lysates obtained from cultured hippocampal neurons infected with Lv-Chili or Lv-Chili-shAP2b1 on DIV4 for 6 days. **d** Results of quantitative WB analysis. *ns*, nonsignificant; *p < 0.05, **p < 0.01 (one-sample *t* test). Number of independent experiments (*N*): GluA2 (4), GluA1 (4), and TrkB (3). *Error bars* indicate SEM

The experiments described above suggested that AP2b1 knockdown lowers total GluA2 level in developing neurons. To confirm that, the total amount of GluA2 was determined by GluA2 immunostaining in hippocampal neurons with AP2b1 knockdown and subsequent analysis revealed an approximately 50 % reduction (Fig. 4a, b). This observation was further biochemically confirmed by quantitative Western blot (qWB) in neurons that were transduced on DIV4 for 6 days with control lentiviral vector Lv-Chili or an shAP2b1-carrying vector (Fig. 4c, d). At the same time, GluA1 and TrkB levels slightly increased in neurons with AP2 knockdown compared with control cells (Fig. 4c, d).

### GluA2 Is Needed for Proper AP2b1-Dependent Dendritic Arbor Morphology

**Fig. 5:**
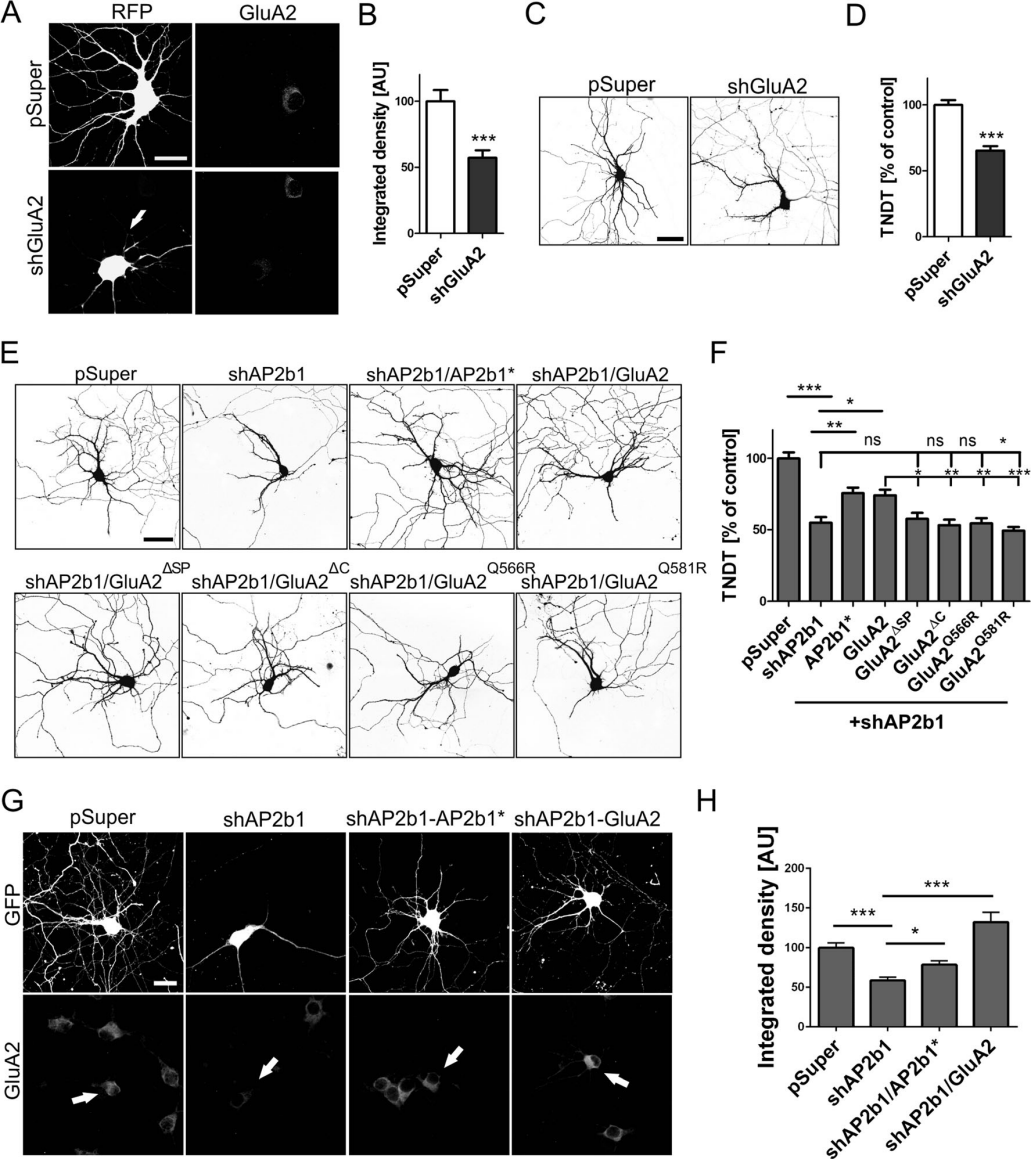
GluA2 is required for AP2b1-dependent proper dendritic arbor morphology. **a** Representative confocal images of cultured hippocampal neurons transfected (*arrows*) on DIV7 for 3 days and immunofluorescently stained as indicated. *Scale bar* = 20 μm. **b** Quantitative analysis of total level of GluA2 immunofluorescence in neurons transfected as in **a**. The integrated density of GluA2 immunofluorescence in transfected cells (cell body) was normalized to that in non-transfected neurons. The data are expressed as a mean value normalized to control. *Error bars* indicate SEM. ***p < 0.001 (Mann-Whitney test). Cell images were obtained from four independent culture batches. Number of cells per variant (*n*): pSuper (32) and shAP2b1 (32). **c** Representative confocal images of cultured hippocampal neurons transfected on DIV7 for 3 days as indicated. Neuron morphology was visualized by co-transfection with GFP-encoding plasmid. *Scale bar* = 50 μm. **d** Total number of dendritic tips (TNDT) of neurons transfected as in **c**. The data are expressed as a mean value normalized to control. *Error bars* indicate SEM. ***p < 0.001 (Mann-Whitney test). Cell images were obtained from four independent culture batches. Number of cells per variant (*n*): pSuper (40) and shGluA2 (40). **e** Representative confocal images of cultured hippocampal neurons transfected on DIV7 for 3 days as indicated. The GFP-encoding plasmid was co-transfected to visualize neuron morphology. *Scale bar* = 50 μm. **f** TNDT of neurons transfected as in **e**. The data are expressed as a mean value normalized to control. *Error bars* indicate SEM. *ns*, nonsignificant; ***p < 0.001, **p < 0.01, *p < 0.05 (one-way ANOVA test followed by Bonferroni multiple-comparison test). Cell images were obtained from three independent culture batches. Number of cells per variant (*n*): all variants (30), except from pSuper (29). **g** Representative confocal images of cultured hippocampal neurons transfected (*arrows*) on DIV7 for 3 days and immunofluorescently stained as indicated. *Scale bar* = 20 μm. **h** Quantitative analysis of total level of GluA2 immunofluorescence in neurons transfected as in g. The integrated density of GluA2 immunofluorescence in transfected cells (cell body) was normalized to that in non-transfected neurons. The data are expressed as a mean value normalized to control. *Error bars* indicate SEM. *p < 0.05, ***p < 0.001 (Kruskal-Wallis test followed by Dunn’s multiple-comparison test). Cell images were obtained from three independent culture batches. Number of cells per variant (*n*): pSuper (32), shAP2b1 (31), shAP2b1/AP2b1* (30), and shAP2b1/GluA2 (29)

The results described to this point indicate that AP2 is needed for the proper dendritic branching of hippocampal neurons and the proper cellular level of GluA2. Earlier studies showed that AMPAR subunits are needed for the dendrite growth of optic tectal neurons [19] and cortical neurons [18]. However, no data have been reported for hippocampal neurons. Therefore, we first determined whether GluA2 knockdown phenocopies the effects of AP2 knockdown. Transfection of DIV7 hippocampal neurons for 3 days with pSuper-shGluA2 [30] caused ∼50 % GluA2 level decrease and significant TNDT reduction (Fig. 5a–d), confirming the contribution of GluA2 to the proper dendritic arbor morphology of developing hippocampal neurons.

Next, we examined whether the negative effect of AP2b1 knockdown on dendritic trees could be rescued by GluA2 overexpression. DIV7 hippocampal neurons were transfected with shAP2b1#2 together with wild-type GluA2. We also used four mutants of this AMPAR subunit, which (*i*) could not be delivered to the cell surface (GluA2-ΔSP), (*ii*) could not stabilize at the cell surface (GluA2-ΔC), and (*iii*) could not act as an active ion channel (GluA2-Q566R and GluA2-Q581R). Cells that were transfected with pSuper, shAP2b1#2 alone, or shAP2b1#2 in combination with an AP2b1-shRNA-resistant construct (EGFP-AP2b1*) served as additional controls. Three days posttransfection, the neurons were fixed, and the TNDT was assessed (Fig. 5e, f). Consistent with the previous experiments, cells that were transfected with shAP2b1 alone exhibited a significant reduction of the TNDT. The co-transfection of shAP2b1 and EGFP-AP2b1* substantially, although not completely, reversed the shAP2b1-induced simplification of dendritic arbors (Fig. 5e, f) as well as GluA2 levels (Fig. 5g, h). Phenotypic rescue was achieved almost to the same extent when shAP2b1 was co-transfected with a plasmid that encoded wild-type GluA2 (Fig. 5e–h). However, no rescue was observed when shAP2b1 shRNA was instead co-transfected with GluA2 mutants. Altogether, the data showed that AP2b1-dependent dendritic tree morphology deficits were rescued only by the addition of GluA2 with intact ion channel, which could be delivered to the cell surface (Fig. 5e, f).

### Lack of AP2b1 Does Not Affect Lysosomal Targeting and Degradation of GluA2

**Fig. 6:**
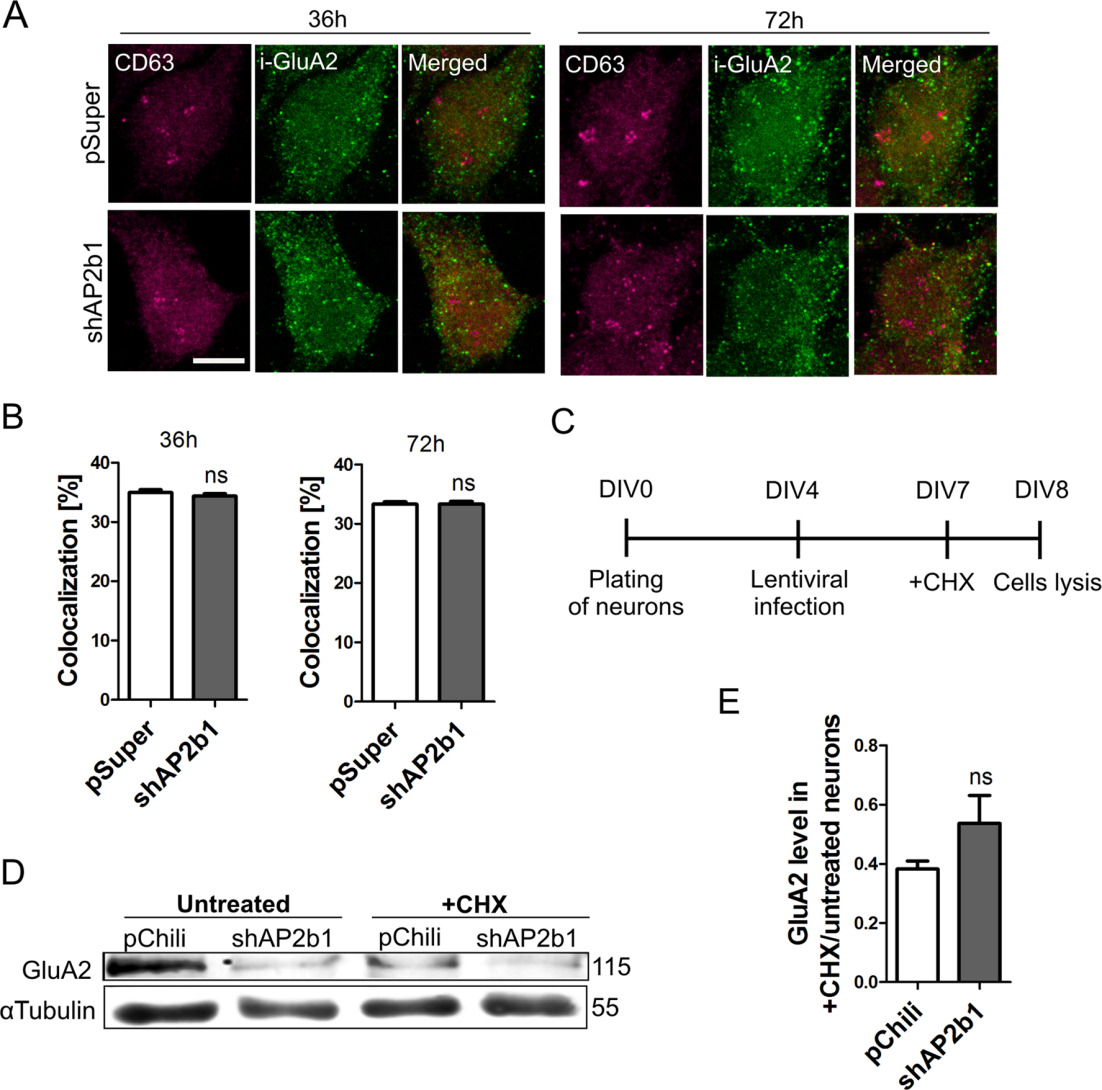
Lack of AP2b1 does not influence lysosomal targeting and degradation of GluA2. **a** Representative confocal images of cultured hippocampal neurons transfected as indicated, immunofluorescently stained for pool of internalized GluA2 (i-GluA2, *green*) and lysosomal marker CD63 (*magenta*). *Scale bar* = 5 μm. **b** Results of colocalization analysis of i-GluA2 with CD63 in the cell soma of neurons transfected and treated as in **a**. The data are expressed as a mean value normalized to control. *Error bars* indicate SEM. *ns*, nonsignificant (Mann-Whitney test). Cell images were obtained from three independent culture batches. Number of cells per variant (*n*): pSuper (26) and shAP2b1 (28) for 36 h; pSuper (30) and shAP2b1 (29) for 72 h. **c** The time-course of the experiment. **d** Representative Western blot showing GluA2 levels in protein extracts obtained from cultured hippocampal neurons infected with Lv-Chili (control) or Lv-Chili-shAP2b1 on DIV4 for 4 days and treated as indicated in **c**. **e** Results of qWB analysis of GluA2 degradation in cells treated as in **c**. The data are expressed as a ratio of GluA2 levels in CHX-treated neurons to untreated cells. GluA2 levels were normalized to tubulin. *ns*, nonsignificant (Mann-Whitney test). Number of independent experiments *N* = 3. *Error bars* indicate SEM

The results described above led us to conclude that the presence of AP2b1 is critical for maintaining the amount of GluA2 that is needed for dendritic arborization. The cellular level of a given protein is an effect of its biosynthesis and degradation. Based on the experiments that were performed so far, we could not determine which of these processes is affected in the case of GluA2 upon AP2b1 knockdown. To determine whether the enhanced degradation of GluA2 occurs upon AP2b1 knockdown, we investigated the amount of internalized GluA2 that reached lysosomes, which are responsible for GluA2 degradation [45]. DIV7 neurons were transfected with pSuper or shAP2b1#2 for 36 or 72 h, and an internalization assay was then performed analogously to the assay described above, with the additional step of immunofluorescently detecting CD63, a lysosomal membrane glycoprotein (Fig. 6a, b). At both of the tested time points, the analysis of colocalization between internalized GluA2 and CD63 revealed approximately 30–40 % overlap under control conditions. Correction for random colocalization decreased GluA2 and CD63 puncta overlap to 10–15 % (Fig. S1, Online Resource 1). Nevertheless, regardless of method used for analysis, we found no meaningful differences between control and experimental variants. Similar results were obtained for the colocalization analysis of internalized GluA2 with Rab7, a marker of late endosomes (not shown). Therefore, increased GluA2 lysosomal degradation could not account for the robust decrease in GluA2 upon AP2b1 knockdown (Figs. 6b and S1, Online Resource 1).

The above analysis implies that AP2b1 knockdown does not elevate the degradation of GluA2 via the lysosomal pathway. To further confirm this observation and additionally exclude possible GluA2 degradation via alternative systems (e.g., the proteasome; [46, 47]), we compared the rate of GluA2 degradation in control and AP2b1-deficient neurons upon protein synthesis inhibition. To accomplish this, DIV4 neurons were transduced with Lv-Chili or Lv-Chili-shAP2b1. Three days post-infection, the cells were treated with CHX (10 μM; Fig. 6c), and the level of GluA2 was assessed 24 h later by qWB (Fig. 5d). However, upon protein biosynthesis inhibition, we did not observe an increase in the degradation of GluA2, measured as the ratio of GluA2 levels in CHX-treated and untreated cells, in neurons that were transduced with Lv-Chili-shAP2b1 compared with Lv-Chili-infected neurons (Fig. 6e). Thus, we concluded that the lower amount of GluA2 upon AP2b1 knockdown was not attributable to its higher degradation rate.

### Knockdown of AP2b1 Affects GluA2 Levels by Controlling mTORC1 Signaling

**Fig. 7:**
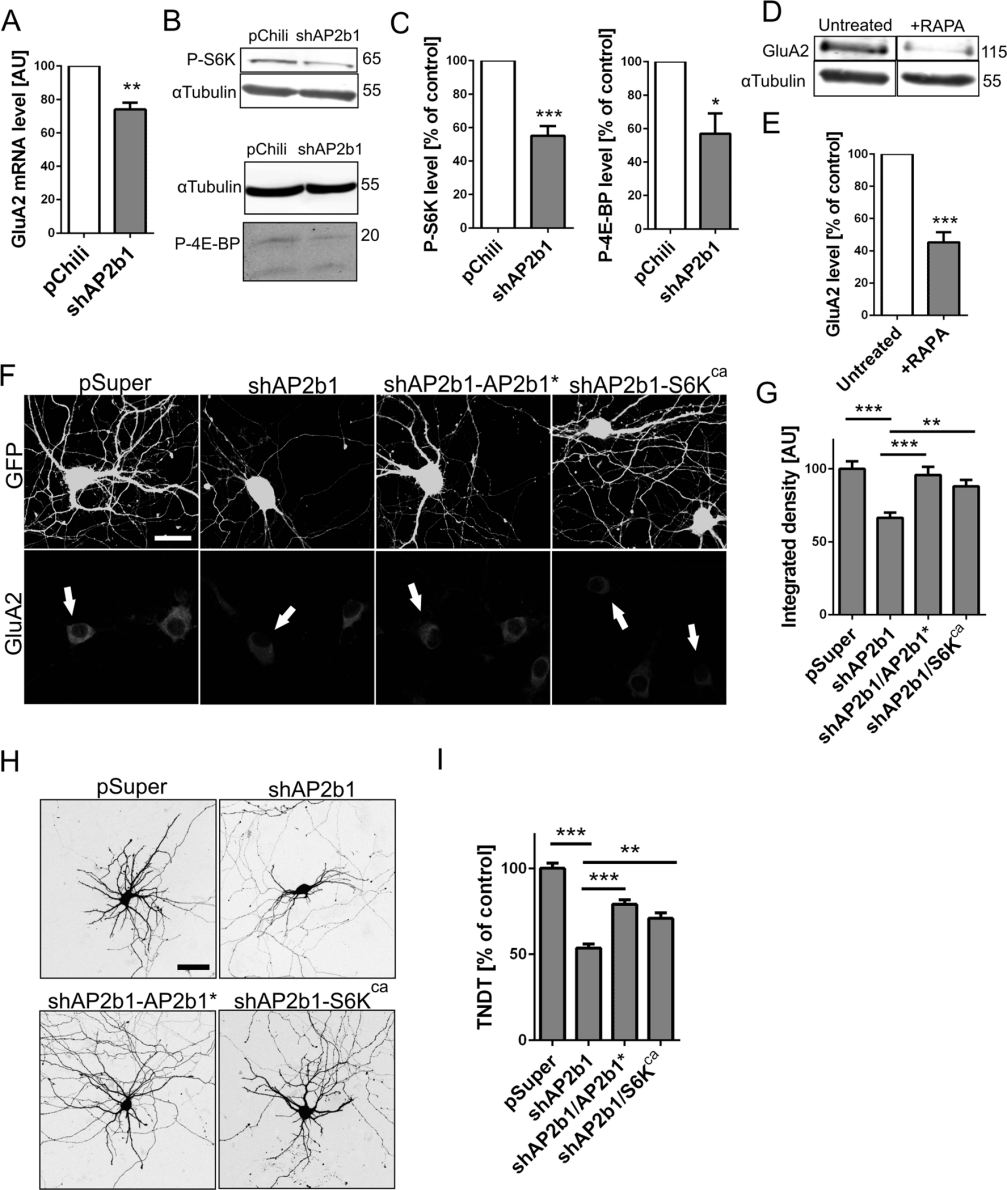
AP2 controls GluA2 levels via mTORC1. **a** GluA2 mRNA levels in cultured hippocampal neurons infected with Lv-Chili (control) or Lv-Chili-shAP2b1 on DIV4 for 6 days. **p < 0.01 (one-sample *t* test). Number of independent experiments *N* = 4. *Error bars* indicate SEM. **b** Representative Western blot showing phospho-S6K (T389) (P-S6K) and phospho-4E-BP1 (T37/46) (P-4E-BP) levels in protein lysates obtained from neurons infected as in **a**. **c** Results of qWB analysis of P-S6K and P-4E-BP levels, normalized to tubulin, in protein lysates from neurons infected as in **a**. ***p < 0.001, *p < 0.05 (one-sample *t* test). Number of independent experiments *N* = 6 (P-S6K) and *N* = 4 (P-4E-BP). *Error bars* indicate SEM. **d** Representative Western blot showing levels of GluA2 in protein extracts obtained from control DIV9 neurons or cells after 2 days rapamycin (RAPA, 100 nM) treatment. **e** Results of qWB analysis of GluA2 levels, normalized to tubulin, in protein extracts of cells treated as in **d**. ***p < 0.001 (one-sample *t* test). Number of independent experiments *N* = 5. *Error bars* indicate SEM. **f** Representative confocal images of cultured hippocampal neurons transfected on DIV7 for 3 days as indicated. Cells were immunofluorescently stained for GluA2. GFP was co-transfected to identify transfected neurons (*arrows*). *Scale bar* = 20 μm. **g** Quantitative analysis of total levels of GluA2 immunofluorescence in neurons transfected as in **f**. The integrated density of GluA2 immunofluorescence in cell bodies of transfected cells was normalized to that in non-transfected neurons. The data are expressed as a mean value normalized to control. *Error bars* indicate SEM. ***p < 0.001, **p < 0.01 (Kruskal-Wallis test followed by Dunn’s multiple-comparison test). Cell images were obtained from four independent culture batches. Number of cells per variant (*n*): pSuper (39), shAP2b1 (46), shAP2b1/AP2b1* (40), and shAP2b1/S6K^ca^ (42). **h** Representative confocal images of cultured hippocampal neurons transfected on DIV7 for 3 days as indicated. *Scale bar* = 50 μm. **i** Total number of dendritic tips (TNDT) of neurons transfected as in **h**. The data are expressed as a mean value normalized to control. *Error bars* indicate SEM. ***p < 0.001, **p < 0.01 (Kruskal-Wallis test followed by Dunn’s multiple-comparison test). Cell images were obtained from five independent culture batches. Number of cells per variant (*n*): pSuper (50), shAP2b1 (50), shAP2b1/AP2b1* (47), and shAP2b1/S6K^ca^ (47)

Because degradation was not the reason for the reduction of GluA2 levels due to AP2b1 knockdown in neurons, we investigated whether the lower amount of functional AP2 affects GluA2 expression. RT-qPCR analysis of GluA2 mRNA levels in hippocampal neurons that were infected with Lv-Chili and Lv-Chili-shAP2b1 revealed a significant, although relatively mild, decrease in GluA2 mRNA upon AP2b1 knockdown (Fig. 7a). This suggests that the observed reduction of GluA2 was at least partially explained by the lower availability of mRNA.

The inhibition of CME downregulates PI3K and AKT [11], which are widely known activators of mTORC1, an important regulator of protein translation. Therefore, we reasoned that in our experimental model, AP2 deficiency led to the inhibition of mTORC1 activity and a further decrease in GluA2 levels. To test this hypothesis, we infected DIV4 neurons with an Lv-Chili control vector or Lv-Chili-shAP2b1. Six days later, upon AP2b1 knockdown, the levels of phosphorylated forms of two canonical mTORC1 substrates: p70 ribosomal S6 protein kinase 1 (S6K) and eukaryotic translation initiation factor 4E-binding protein 1 (4E-BP1) decreased nearly by 50 % (Fig. 7b, c).

Because AP2b1 deficiency negatively affected mTORC1 activity, we next investigated whether mTOR inhibition impacts GluA2 levels. DIV7 hippocampal neurons were treated with rapamycin (100 nM) for 2 days, and qWB analysis of GluA2 protein abundance was performed (Fig. 7d). The results revealed that rapamycin treatment significantly decreased the level of GluA2 compared with control (Fig. 7e), indicating that mTOR signaling is indeed involved in regulating GluA2 levels in developing neurons. It should be noted, however, that mTORC1 inhibition, unlike AP2b1 knockdown, decreased also GluA1 and TrkB levels (Fig. S2, Online Resource 1). Nevertheless, in light of this finding, we further verified whether mTORC1 mediates the impact of AP2b1 deficiency on GluA2. Thus, we tested whether the negative effects of shAP2b1#2 on GluA2 expression and dendritic trees can be rescued by the constitutively active form of S6K (S6K1T389E [S6K^ca^]), the activity of which is resistant to mTOR inhibition. DIV7 hippocampal neurons were transfected with shAP2b1#2 together with S6K^ca^. Cells that were transfected with pSuper, shAP2b1#2 alone, or shAP2b1#2 in combination with AP2b1* served as additional controls. As shown in Fig. 7f, g, the overexpression of S6K^ca^, similar to AP2b1*, was able to counteract the shAP2b1#2-induced decrease in GluA2 immunofluorescence. Moreover, the partial rescue of the TNDT was also achieved when shAP2b1 was co-transfected with a plasmid that encoded S6K^ca^ (Fig. 7h, i). Thus, we concluded that AP2 controlled GluA2 levels and dendritogenesis via mTOR-S6K1 signaling.

## Discussion

In the present study, we demonstrated that the AP2 adaptor complex, a critical component of the mechanism of CME, is needed for the proper dendritic arbor morphology of hippocampal neurons in vitro and in vivo. AP2 knockdown concomitantly decreased dendritic arborization and the level of the GluA2 subunit of AMPARs. Consequently, supplementation of functional GluA2 rescued dendritic arbor simplification that was caused by AP2b1 knockdown. Moreover, we found that AP2 deficiency also reduced the activity of mTOR, a positive regulator of GluA2 synthesis. Accordingly, overexpression of the constitutively active form of the mTOR downstream effector S6K1 in AP2-deficient neurons rescued GluA2 expression and dendritic morphology deficits. Altogether, our findings demonstrate that AP2 controls dendritogenesis by ensuring suitable levels of GluA2 synthesis.

### AP2 Contributes to Proper Neuronal Morphology of Mammalian Neurons

An important finding of the present study was that the AP2 complex is required for the proper dendritic morphology of rat hippocampal neurons, both cultured in vitro and developing in vivo. Thus far, two cellular functions of AP2 have been described. AP2 plays a canonical role during CME, when AP2 serves as an endocytic adaptor that links cargo with the clathrin coat [5]. Recently, however, the contribution of AP2 to autophagosome formation and the degradation of selected proteins has been described [48]. Data from *Drosophila* suggest that disturbances in endocytosis of the adhesion molecule Nrg that are caused by AP2 deficiency indeed lead to the simplification of dendritic arbors [12]. These data imply that the AP2-dependent trafficking of selected membrane proteins contributes to dendritogenesis but it remains to be established, disturbance of which specific cargo trafficking leads to dendritic growth and GluA2 synthesis inhibition upon AP2 knockdown in mammalian neurons. The list of potential candidates is quite long (e.g., TrkB, cholesterol receptors, neurotransmitter receptors). In our opinion, it will be very challenging to pinpoint specific AP2 cargo, the disrupted trafficking of which induces changes that are specific to AP2 knockdown. Also we cannot exclude that the observed phenotypic effects of AP2 deficiency are not linked to inhibition of specific cargo CME but rather stem from overall change in protein expression and/or cell metabolism resulting from global change of CME. This could be potentially corroborated by the observation that AP2b1 knockdown lowers activity of mTORC1, a known regulator of cellular metabolism. Yet, we show that lowering mTORC1 activity has broader effect on protein synthesis than AP2b1 knockdown, which argue against global changes and implies existence of more specific, yet undiscovered, mechanism.

### AP2 Controls Dendritic Arborization by Regulating GluA2 Biosynthesis

The contribution of GluA2 to dendritogenesis of developing tectal and cortical neurons has been described [18, 19]. Several studies suggest that in mature neurons AMPARs are internalized via CME in response to increased neuronal activity [7–9, 44]. In fact, upon LTD induction, AMPAR internalization requires interactions with AP2 [9]. In unstimulated cells, however, AMPAR endocytosis is relatively low and is CME- and AP2-independent [43, 44]. But rules of GluA2 trafficking in developing neurons have not been studied neither under basal conditions nor upon stimulation. Therefore, we were not able to exclude that under our experimental conditions AP2b1 knockdown leads to disturbance of GluA2 internalization. Therefore, we studied basal GluA2 endocytosis in young neurons. Our results clearly showed that, similar to unstimulated mature neurons, young neurons that were undergoing intensive dendritogenesis did not require AP2 for GluA2 internalization. However, additional experiments, results of which are presented in Fig. S3, revealed that in developing neurons standard protocols used to induce internalization of AMPARs are inefficient, suggesting that indeed some aspects of AMPAR trafficking differ between developing and mature neurons.

Although we did not observe effects of AP2b1 knockdown on GluA2 internalization in developing neurons, we found that AP2b1 is required to sustain the cellular levels of GluA2 that are sufficient for dendritic growth and/or maintenance. This conclusion was corroborated by several observations. First, we found that AP2b1 knockdown decreased the cellular level of GluA2. Second, GluA2 knockdown, similar to AP2 deficiency, simplified dendritic arbors. Third and most importantly, supplementation with functional GluA2 restricted the dendritic arbor phenotype that was caused by AP2 deficiency. These observations raise the issue of the way in which AP2 controls GluA2 expression. Our data argue for the lower level of GluA2 originating from its disrupted biosynthesis. First, we found a slight decrease in the amount of GluA2 mRNA upon AP2b1 knockdown. Second, our data imply that AP2 controls GluA2 synthesis also at the level of translation. Still, however, an open question remains how lower GluA2 expression translates to decreased complexity of dendritic arbor. Hippocampal AMPARs most often work as heterodimers (i.e., GluA1-GluA2, GluA2-GluA3), and we showed that overexpression of fully functional GluA2 was capable to rescue dendritic defects caused by AP2b1 knockdown. This could mean that decreased number of dendrites stems from lowered AMPAR-dependent transmission as both heterodimer types should be affected by AP2b1 knockdown. However, in certain circumstances, AMPAR subunits form homodimers (e.g., GluA1-GluA1) [49]. Thus, it is possible that upon AP2b1 knockdown cells start to form GluA1 homodimers to sustain AMPAR-dependent transmission. Such switch could in turn affect dendritic calcium signaling, which has been repeatedly shown to impact dendritogenesis either “positively” or “negatively” depending on its entry route [50]. Moreover, it was demonstrated in cortical pyramidal neurons that overexpression of GluA1 (which likely favors formation of GluA1 homodimers) was not effective in inducing dendritic arborization while GluA2 overexpression stimulated dendritogenesis quite potently [18]. Thus, further experiments are needed to distinguish direct and indirect effects of GluA2 deficiency on dendritic branching.

### AP2 Controls GluA2 Levels via the mTORC1 Pathway

mTORC1 controls protein translation via its downstream effectors 4E-BP1 and p70S6K. In neurons, mTORC1 is involved in controlling protein synthesis in response to BDNF, insulin, and a variety of neurotransmitters [25]. mTOR, 4E-BP1, and p70S6K also regulate dendritic arbor growth [20, 21]. Considering the previous findings and new evidence that was provided by our study, we propose that AP2 controls GluA2 synthesis via the mTORC1 pathway. Several findings in the present study support this hypothesis. First, AP2b1 knockdown decreased mTORC1 pathway activation. Second, mTOR inhibition by rapamycin lowered GluA2 protein levels. Third, overexpression of the active form of p70S6K1 counteracted the decrease in the cellular content of GluA2 and dendritic arbor simplification that were induced by AP2b1 loss. However, AP2 knockdown, in contrast to rapamycin treatment, decreased the cellular levels of GluA2 but not the levels of GluA1 or TrkB. How can the effect of AP2b1 knockdown be so specific? In our opinion, additional means of the regulation of protein synthesis must be considered. For example, GluA2 protein translation was predicted and subsequently shown to be regulated by a specific set of microRNAs [51, 52]. One of them is miR124a, which does not recognize, for example, GluA1 [53]. Our preliminary data, however, ruled out possibility that miR124a expression increases in neurons upon AP2 knockdown (data not shown). Therefore, future work should address how, in addition to mTORC1 regulation, AP2 controls the expression of GluA2 so specifically and whether microRNAs other than miR124a may be involved.

In summary, we found evidence that the AP2 adaptor complex is needed for the dendritogenesis of mammalian neurons, and mTOR-dependent GluA2 biosynthesis was a contributing mechanism.

## Electronic supplementary material

ESM 1 (PDF 65 kb)
