# Supplementary material for: Adaptor Complex 2 Controls Dendrite Morphology via mTOR-Dependent Expression of GluA2
Source: Mol Neurobiol. 2017 Feb 11;55(2):1590–606. doi: 10.1007/s12035-017-0436-3 (PMC5820378; doi:10.1007/s12035-017-0436-3)
Supplement: Supplementary file 1 — (PDF 65 kb) [file 12035_2017_436_MOESM1_ESM.pdf]

Koscielny et al. - Online Resource 1. Supplementary Figures

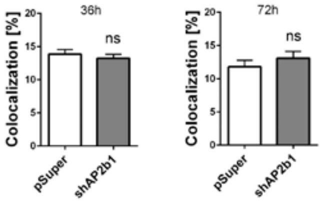

Figure S1. Results of colocalization analysis of i-GluA2 with CD63 in the cell soma of neurons transfected and treated as in Fig. 6*A, B* after inclusion of randomization control. The data are expressed as a mean value normalized to control. Error bars indicate SEM. ns, non significant. Cell images were obtained from three independent culture batches. Number of cells per variant (n): pSuper (18) and shAP2b1 (26) for 36 h; pSuper (21) and shAP2b1 (20) for 72 h.

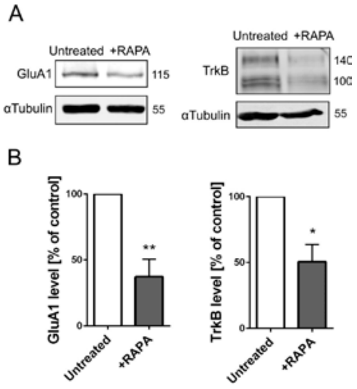

Figure S2. ***A,*** Representative Western blots showing levels of GluA1 and TrkB in protein extracts obtained from control DIV9 neurons or cells after 2 days rapamycin (RAPA, 100 nM) treatment. ***B,*** Results of quantitative WB analysis of GluA1 and TrkB levels, normalized to tubulin, in protein extracts of cells treated as in *A*. $*p < 0.05$, $**p < 0.01$ (one-sample t-test). Number of independent experiments (N): GluA1 (5), TrkB (4). Error bars indicate SEM.

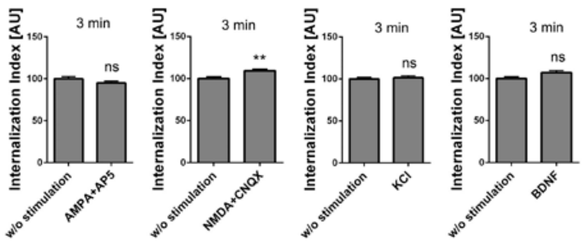

Figure S3. Quantitative analysis of GluA2 internalization index in cultured hippocampal neurons at DIV10, untreated (w/o stimulation) or treated with 50 µM AMPA+50 µM AP5, 50 µM NDMA+50 µM CNQX, 90 mM KCl or 100 nM BDNF for indicated time points. The internalization index was calculated using the following formula: i-GluA2 / s-GluA2 + i-GluA2. The results are presented as a mean value normalized to control ± SEM. ns, nonsignificant; **$p < 0.01$; (Mann Whitney test). Cell images were obtained from two independent culture batches. Number of cells per variant (n): 20.
